# Excess secondary sludge reuse by H_2_O_2_ thermal dehydration

**DOI:** 10.1007/s11356-024-32568-8

**Published:** 2024-02-28

**Authors:** Ana Udaondo, Vicente Montes, Olga Gimeno, Francisco Javier Rivas

**Affiliations:** 1 Departamento de Ingeniería Química y Química Física, Instituto Universitario de investigación del agua, cambio climático y sostenibilidad, Universidad de Extremadura, Badajoz, Spain; 2 Departamento de Química Orgánica e Inorgánica, Instituto Universitario de investigación del agua, cambio climático y sostenibilidad, Universidad de Extremadura, Badajoz, Spain

**Keywords:** Wastewater treatment, Sludge management, Hydrogen peroxide, Adsorbent, Thermal treatment, Circular economy

## Abstract

The excess of activated sludge generated in municipal wastewater treatment plants constitutes one of the challenging problems facing modern society. The high-water content of this waste makes difficult the transport, disposal, and management of these solids. In this work, activated sludge excess from a secondary clarifier has been dehydrated by means of a combination of temperature and hydrogen peroxide treatment. Three main operating variables have been considered to affect sludge dewaterability and filterability. Temperature (120–180 °C), hydrogen peroxide dose (0.01–0.03 M), and treatment time (20–60 min) influence have been assessed by completing a 15-run Box Behnken experimental design. Different output variables (water content, resistance to filtration, sedimentation volumetric index, extracellular polymeric substances, etc.) have been monitored. Generally, temperature seems to be the most influencing parameter to obtain a dehydrated sludge with acceptable management/disposal characteristics (sludge volume reduction and filterability). In line with the concept of circular economy, an attempt has been conducted to obtain a sustainable biosorbent from the dehydrated sludge generated in the previous stage. Optimum conditions of carbonization and activation revealed that the solid obtained at 400 °C by using ammonium nitrate as activation agent was the most efficient absorbent to eliminate some model compounds from water (namely, phenol, ofloxacin, and diuron); however, a clear improvement margin in the synthesis is foreseen.

**Supplementary Information:**

The online version contains supplementary material available at 10.1007/s11356-024-32568-8.

## Introduction

Regardless of the development level of the regions, wastewater treatment is manifestly increasing in most countries. However, the fight against water scarcity creates a new challenge: how to manage sewage sludge. As cleaner water is increasingly discharged into natural receiving media, huge amounts of sewage sludge are produced (Mateo-Sagasta et al. 2015). With the exception of some particular countries, sewage sludge generation in Europe has not substantially changed for the last decade (2010–2019), amounting around 10–11 thousand tons in a group of 34 countries excluding Russia (http://appsso.eurostat.ec.europa.eu/, 2023). Contemporary environmental policies advocate for paradigm shifts in waste management, aiming to achieve zero waste generation. This entails either the elimination of waste production altogether or the transformation and recycling of waste into valuable products. Potential reuse of sewage sludge is conditioned by the presence of heavy metals, toxic organic contaminants, and biological hazards. Main sewage sludge treatment methods vary within the EU, as fertilizer for agriculture, composting, incineration, or landfilling. Some statistics can be found in Eurostat (http://appsso.eurostat.ec.europa.eu, 2023).

In any case, whether the sludge is reused or disposed of, this subproduct must be managed, transported, or stored. Sewage sludge mainly contains water, organic and inorganic substances (toxic or harmless, including heavy metals), microorganisms, and in some cases large particles such as sand or grit. Accordingly, sewage can be characterized based on the physical properties (indicating the easiness of manipulation and handling), chemical composition (indicative of the presence of toxic substances), and biological nature (revealing the existence of pathogens) (Grobelak et al. 2019).

Any strategy applied to sludge treatment must consider the elimination of biological and chemical hazards and volume reduction (dewatering). The latter is considered of paramount importance in any sewage sludge treatment process. Dewatering is directly connected to volume reduction and, consequently, to the costs associated with transport. Moreover, sludge dewatering facilitates the further processing of this material (Kamizela and Kowalczyk 2019). Dewatering of sludge is performed after conditioning by either chemical, mechanical, thermal, and/or unconventional processes. Each strategy presents advantages and drawbacks. All of them can be included in one of the two categories: those affecting floc hydrophilicity by extracellular polymeric substance disruption or, alternatively, processes that modify sludge floc compressibility and particle size (Chen et al. 2022).

Thermal treatments (belonging to the first category) have been demonstrated to significantly improve sludge dewaterability (Cao et al. 2021; Liu et al. 2019; Yu et al. 2014; Zikakis et al. 2019). High temperature application, however, may result in an excessive energy consumption and generation of refractory end products that accumulate in the water bulk of the filtrate (Chen et al. 2022). Some alternative strategies have, therefore, been adopted to reduce the required temperatures. Hence, combination of thermal processes in the presence of chemicals (oxidants or not) has emerged as a new investigation field in sludge conditioning.

Substances like persulfate (Chen et al. 2021; Ruan et al. 2021), peroxymonosulfate (Li et al. 2018), hydrogen peroxide (Abelleira et al. 2012b), CaCl_2_ (Guan et al. 2012; Yu et al. 2014), cations, acids, and alkalis (Neyens and Baeyens 2003) have been used in the past. In the particular case of hydrogen peroxide, this oxidant has preferentially been used in Fenton like systems at ambient temperature (Ferrentino et al. 2020; Liu et al. 2022b; Yu et al. 2019; Yuan et al. 2018). Surprisingly, the number of studies using the thermal decomposition of H_2_O_2_ in sewage sludge dewatering is rather low (Abelleira et al. 2012b; Cacho Rivero and Suidan 2006; Genç et al. 2002b; Takashima and Tanaka 2008; Zhang et al. 2020). In most of the bibliographic reports, the final objective was the improvement of sludge characteristics to be further anaerobically treated. Abelleira et al. reported the benefits of adding H_2_O_2_ to a preheated reactor by injecting steam as heat source (Abelleira et al. 2012b; Abelleira et al. 2012a). These authors completed an experimental series based on the design of experiments with temperature, reaction time, and H_2_O_2_ dose as the main influencing variables.

In addition to the dewatering process, in line with circular economy, transformation of wastes to valuable products is highly recommended. In this sense, the production of sludge-based adsorbents for the elimination of contaminants in water has been described in specialized literature. This strategy seeks a dual purpose of solid waste reuse and pollution remediation, according to the aforementioned concept of circular economy and carbon neutrality (Hu et al. 2022). Sewage sludge is a carbon-rich, sustainable, and abundant resource that can be purchased at a low price and used to generate ecofriendly adsorbents. The absorbents are applied to remove a range of pollutants from air and water (Soffian et al. 2022).

Various methods have been employed to synthesize the biomass-derived activated carbon, for example, direct pyrolysis, physical activation, chemical activation, physical–chemical activation, and microwave activation (Bian et al. 2018). As a rule of thumb, the S_BET_ of sludge-derived activated carbons is low due to the high percentage of residual metals. Therefore, many studies have been focused on enhancing the carbonization conditions in order to obtain a higher S_BET_. Carbonization temperature has been proved as one of the most important factors influencing the characteristics of synthesized absorbents. Activation conditions (comprising activation type, agent, and temperature) also affect the porosity of the biomass-derived solids (Gong et al. 2020).

Municipal sewage sludge has been used as a raw material to produce carbon-based adsorbents for the adsorption of several contaminants in water such as heavy metals (Koetlisi and Muchaonyerwa 2019; Ngambia et al. 2019; Rezai and Allahkarami 2021), dyes (Chen et al. 2021), pesticides (Sanz-Santos et al. 2022; Zhang et al. 2021), phenols (Kou et al. 2021), polycyclic aromatic hydrocarbons (Godlewska et al. 2019), and pharmaceuticals (Ding et al. 2012; Yin et al. 2019). Also, some works in the literature report the use of activated sludge as a raw substance to produce biochar for CO_2_ capture (Liu et al. 2022a; Liu et al. 2022b).

Taking into account the previous logic sequence: excess sludge generation → management difficulties due to water content → necessity of sludge dehydration → new trends in waste recycling/reuse, in this work, a slightly different methodology to that reported by Abelleira and coworkers (Abelleira et al. 2012b; Abelleira et al. 2012a) was adopted. In this study, hydrogen peroxide was injected and mixed with the sludge once the temperature of the media was achieved. Temperature highly influences H_2_O_2_ decomposition. This parameter accelerates H_2_O_2_ homolytic excision to generate hydroxyl radicals, however, also favoring the inefficient peroxide decomposition to oxygen and water due to high local concentration of radicals that recombine (Rodríguez et al. 2004). Subsequently, the influence of the heating period has been eliminated and the instantaneous effect of temperature has been evaluated. Additionally, the effect of H_2_O_2_ dosage and reaction time has also been investigated by conducting a series of experiments according to a Box Behnken design.

Next, after the sludge dewatering process, two types of adsorbents were synthetized and tested in the adsorption of phenol, ofloxacin, and diuron.

Various strategies for biomass conversion into biosorbents exist, encompassing physical, chemical, or mechanical approaches (Osman et al. 2023). Each method carries distinct advantages and drawbacks. Determining the optimal preparation conditions is inherently experimental, and predicting the best strategy relies on prior empirical experience. This study initiates preliminary experiments to convert dehydrated sludge into a viable biosorbent. The pyrolysis of the raw dehydrated material results in biochar, avoiding the high temperatures necessary for synthesizing activated carbon (Osman et al. 2023). While the term “biochar” typically excludes an activation stage, this study, a priori, contemplates the potential enhancement of biochar performance through the incorporation of an activation stage. Accordingly, adsorbents were obtained after carbonization at 400 °C and 700 °C and activation with KOH and ammonium nitrate.

## Experimental

### Secondary sludge from wastewater treatment plant

Sludge used in this work was collected from the activated sludge purge pipe attached to the secondary settling tank of the municipal wastewater treatment plant located in Badajoz (Southwest Spain). Sludge was stored in plastic containers at 4 °C to reduce activity. Main characteristics of the sludge are shown in Table 1. In this table, the following parameters were calculated.

**Table 1:** Box Behnken experimental design. Coded variables in parentheses

Run (H_2_O_2_, *T*, time)	*W*_c_ (%)	TS (g L^−1^)	VSS (g L^−1^)	SVI_30_ (mL g^−1^)	TtF (s)	WB_1200_ (%)	WB_2000_ (%)	WB_3000_ (%)	RSF (× 10^−12^) (m kg^−1^)
Raw sludge	98.2	19.6	12.4	–	313	3.3	3.5	3.8	8.5
1 (0,0,0)	96.9	13.2	8.4	32.2	312	4.7	5.2	5.5	18.3
2 (0,1,1)	90.6	11.7	3.8	9.5	10	11.7	11.5	12.0	0.1
3 (− 1,0, − 1)	97.8	14.6	6.5	46.2	390	7.0	6.6	7.8	21.3
4 (0,1, − 1)	94.7	14.5	5.5	19.0	88	8.2	8.1	8.1	0.5
5 (1,0, − 1)	92.4	17.0	7.2	13.2	74	6.3	6.7	6.8	1.6
6 (1,0,1)	94.5	12.3	4.7	18.3	68	9.9	10.4	11.1	3.9
7 (1, − 1, − 1)	–	11.6	5.9	–	56	9.1	9.2	9.8	4.4
8 (− 1,0,1)	–	23.2	10.4	–	410	4.6	4.8	5.0	14.6
9 (0,0,0)	–	19.4	8.4	–	784	5.8	5.8	6.4	37.0
10 (0, − 1,1)	98.2	18.1	8.3	55.2	995	5.4	5.8	6.4	37.7
11 (0,1, − 1)	97.8	9.7	4.4	45.1	1088	12.3	12.9	13.4	62.4
12 (− 1,1,0)	94.9	15.2	5.2	19.7	85	9.0	8.9	9.1	1.9
13 (1,1,0)	93.1	17.3	7.8	14.5	18	6.0	6.1	6.5	0.1
14 (− 1, − 1,0)	97.8	22.2	13.5	45.0	1740	3.0	3.6	3.8	58.6
15 (0, − 1, − 1)	95.3	19.8	12.6	21.5	180	4.4	4.6	4.6	11.8
Run	s-EPS			l-EPS			t-EPS		
	Protein (g L^−1^)	COD (mg L^−1^)	TOC (mg L^−1^)	Protein (g L^−1^)	COD (mg L^−1^)	TOC (mg L^−1^)	Protein (g L^−1^)	COD (mg L^−1^)	TOC (mg L^−1^)
Raw sludge	0.11	72.8	148.2	0.06	40.3	121.5	0.179	128.1	318.3
1 (0,0,0)	1.26	2414.5	6840.8	0.18	149.9	445.0	0.29	37.7	100.1
2 (0,1,1)	1.70	3016.0	6783.1	0.37	191.9	571.6	0.13	35.3	97.0
3 (− 1,0, − 1)	1.21	1940.1	4783.5	0.16	80.3	228.3	0.10	35.8	696.8
4 (0,1, − 1)	1.62	1820.6	8675.6	0.33	239.3	533.5	0.23	114.6	254.2
5 (1,0, − 1)	1.35	2514.3	5940.7	0.34	139.7	448.0	0.18	78.0	191.6
6 (1,0,1)	1.37	2476.3	5427.9	0.16	99.2	254.2	0.12	59.5	211.5
7 (1, − 1, − 1)	1.04	1607.5	3742.9	0.14	78.1	217.6	0.07	27.5	93.9
8 (− 1,0,1)	1.63	4144.1	8577.9	0.29	222.6	533.5	0.17	77.0	232.8
9 (0,0,0)	1.37	3152.3	6807.6	0.15	169.7	463.3	0.09	81.9	225.2
10 (0, − 1,1)	1.04	1858.3	4231.3	0.21	124.2	275.6	0.19	63.3	151.9
11 (0,1, − 1)	0.83	1052.7	2998.2	0.09	33.6	81.7	0.06	16.4	48.2
12 (− 1,1,0)	1.63	3274.1	7418.0	0.26	150.0	394.6	0.16	62.7	182.5
13 (1,1,0)	1.82	4663.2	9286.1	0.44	302.0	724.3	0.17	83.7	205.4
14 (− 1, − 1,0)	1.22	2070.6	4780.8	0.20	98.1	185.5	0.12	55.0	100.1
15 (0, − 1, − 1)	1.57	3459.7	7589.0	0.25	264.0	364.1	0.17	58.4	124.5

Water content (*W*_c_) was calculated by drying the solid layer of the settled sludge after treatment at 110 °C; differences between the initial weight and dry residue were used to calculate this parameter.

Total solids (TS) were determined from the weight of the dry residue obtained from 100 mL of the mixed sludge.

Water bound at different centrifugation rpm (WB_rpm_) was determined after the work of Jin et al. (Jin et al. 2004). This parameter measures the weight of the centrifugated cake at different *G* values comparing this value with the amount of total suspended solids (TSS).

Total suspended solids (TSS) were obtained after filtration and drying of the solid at 105 °C for 24 h. Volatile suspended solids (VSS) were determined after calcination under inert atmosphere of the previous solid at 250 °C in an oven to remove volatile compounds.

Specific resistance to filtration (SRF) was calculated by the following equation (Christensen 1983):

1 $$\text{SRF=2}\frac{{A}^{2}\times \Delta P\times b}{\mu \times {C}_{\text{TS}}}$$

where *A* is the surface filtration, ∆*P* is the pressure drop across the sludge cake, *µ* is the absolute viscosity of the filtrate, *C*_TS_ is the concentration of total solids, and *b* is the slope of the *t*/*V* vs *V* plot (*V* is the volume of filtrate). Using SI units, SRF is expressed in m kg^−1^. A related parameter that avoids the calculation of the slope *b* (which in some cases can mislead the observed results) is the time to filter (Abelleira et al. 2012a). This factor measures the time required to filtrate 50% of the original sample (50 mL) in a Buchner funnel (Resma filter paper 75 g/m^2^) applying a predetermined vacuum (35 kPa).

Extracellular polymeric substances (EPS) were divided into three fractions, soluble (s-EPS), loosely bound (l-EPS), and tightly bound (t-EPS) fractions. Loosely and tightly bound EPS were obtained after thermal extraction (Li and Yang 2007). Steps included the centrifugation of 50 mL of raw sludge for 5 min; the supernatant was catalogued as soluble EPS. The centrifugated solid was next resuspended with water to the initial volume of 50 mL. The solution was heated to 50 °C and agitated for 1 min. Centrifugation for 10 min after stirring allowed to obtain the so-called loosely bound EPS. Tightly bound EPS was determined from the solid of the second centrifugation. This solid was diluted to 50 mL with water and heated to 60 °C for 30 min. Centrifugation of the mixture permitted the determination of t-EPS.

Proteins were analyzed by the Lowry methodology (Hartree 1972) by using bovine serum albumin as standard.

Soluble chemical oxygen demand (COD) was measured after solid centrifugation by Dr. Lange vials once digestion at 150 °C was carried out for 30 min. Soluble total organic carbon (TOC) was determined after solid removal by a Shimadzu TOC 5000A analyzer by directly injecting the supernatant solution. Hydrogen peroxide (35% Panreac) was quantified by the iodometric method. Contribution of the remaining H_2_O_2_ to total COD in the aqueous phase was considered by applying the relationship COD_H2O2_ = a C_H2O2_ + b, were COD_H2O2_ is the chemical oxygen demand due to H_2_O_2_ presence and C_H2O2_ is the iodometric concentration of hydrogen peroxide. In any case, the remaining H_2_O_2_ interference in COD analysis was negligible in all cases.

### Procedure

Experiments were conducted in an isothermal stainless-steel reactor operated in discontinuous mode (Fig. S1). The reactor of 0.45 L of capacity was equipped with mechanical agitation, a H_2_O_2_ injection port, thermocouple, sampling port, and safety valve. Once the reactor was loaded with raw sludge, the vessel was pressurized to 3 bar and the oven was turned on to reach the specific working temperature. Next, the calculated amount of concentrated H_2_O_2_ was injected to the reactor through the injection port by pressurizing the system to 6.0 bar. This pressure was enough to carry out the thermal treatment in the liquid phase.

Experiments were conducted by applying a Box Behnken experimental design with three factors evaluated at three levels, namely, hydrogen peroxide concentration in the range 0.01–0.03 M, temperature (120–180 °C), and reaction time (20–60 min). Conditions are coded as − 1 (0.01 M in H_2_O_2_, 120 °C, 20 min), 0 (0.02 M in H_2_O_2_, 150 °C, 40 min), and 1 (0.03 M in H_2_O_2_, 180 °C, 60 min).

### Biosorbent synthesis and characterization

#### Preparation method

Two types of adsorbents were prepared from dewatered sludge after carbonization at 400 and 700 °C and activation with KOH and ammonium nitrate (NN).

The carbonization process was carried out into a vertical stainless-steel reactor of 4.5 cm diameter, under a nitrogen flowrate of 100 mL min^−1^. Temperature was increased from room conditions at a rate of 10 °C min^−1^, until the final temperature (400 or 700 °C) was reached. The final temperature was maintained for 2 h. The obtained carbonized products were named S_400_ and S_700_, respectively, depending on the final temperature applied.

Chemical activation was carried out by using two types of activation agents, KOH and ammonium nitrate (NN) in a weight ratio activation agent/carbonaceous material 3:1. Briefly, 7.5 g of the activation agent was mixed with 2.5 g of the carbonized dehydrated sludge. Next, 1 mL of ultrapure water was added under vigorous magnetic agitation at 40 °C for 1 h. The mixture was transferred to a ceramic crucible and placed in a horizontal furnace under 100 mL min^−1^ of N_2_ for activation. In the case of KOH activation, temperature was raised from room temperature to 800 °C at a rate of 10 °C min^−1^. When the target temperature was reached, the furnace was switched off. Once the system was cold, the solid was washed with H_2_O several times to remove the excess of the potassium salt. In the case of using NN, the temperature was raised from room temperature to 400 °C. In this later case, no washing step was needed. The synthetized adsorbents were named S_KOH_ and S_NN_.

The surface area of the obtained solids was determined from nitrogen adsorption–desorption isotherms obtained on an Autosorb iQ2-C Series using the Brunauer–Emmett–Teller (BET) method. All samples were degassed to 0.1 Pa at 120 °C and 12 h prior to measurement. Elemental analysis was carried out with a CHNS TruSpec Micro (LECO) elemental analyzer. Wavelength dispersive X-ray fluorescence spectroscopy (XRF) was performed with a S8 Tiger apparatus (Bruker).

#### Biosorbent performance

The adsorption capacity of the adsorbents was tested using phenol, ofloxacin, and diuron as model organic pollutants.

Kinetic adsorption experiments were performed in amber bottles in a thermostatic bath at room temperature. Phenol isotherm was obtained in the same experimental apparatus. Kinetic runs were carried out with 2 ppm of initial concentration in each model pollutant and 1 g L^−1^ of the adsorbent. Samples were steadily withdrawn at different times, filtered, and analyzed.

Determination of organic concentration was performed by a HPLC (High-Performance Liquid Chromatography) Shimadzu UFLC-20AD equipped with a degasser, quaternary pump, automatic injection system, and diode array detector. The analysis was carried out by isocratic elution, using 80–20 (v/v) mixture of acetonitrile-acidified water (0.1% v/v with H_3_PO_4_). The stationary phase was a C18 column (Phenomenex) with a particle size of 5 μm and a pore size of 100 Å, packed in 15 × 0.4 cm columns. Detection was performed by absorbance measurement at 220 nm.

Total organic carbon (TOC) in liquid samples was determined with a Shimadzu TOC-L CSN analyzer coupled to an ASI-V automatic sample injector. The data of TC, IC measurements and consequently the TOC values were registered and automatically processed by a TOC-Control V™ software.

## Results and discussion

### Sewage sludge H_2_O_2_ dewatering

The first part of this work was focused on the sewage sludge conditioning by means of the treatment at high temperature in the presence of hydrogen peroxide. After completing the experimental series, several parameters were evaluated to sustain the suitability and benefits (if any) of the treatment. Results obtained after completing the Box Behnken experimental design are displayed in Table 1 (conditions are compiled in parentheses in coded variables).

#### Solid-water content evolution

At the sight of the results obtained, water content slightly diminished from 98% to values in the proximity of 94–95%. A minimum value of 90% was obtained when 180 °C at 0.02 M in H_2_O_2_ was applied for 60 min. Temperature seems to be the most influential factor in this parameter. By using the Box Behnken experimental design, *W*_c_ was adjusted to a two-factor interaction equation leading to the expression (coded values for variables):

2 $${W}_{\text{c}}\text{ = 95.57}-\text{0.85}\left[{\text{H}}_{2}{{\text{O}}}_{2}\right]-\text{2.19 }T-\text{0.74 }t-\text{0.3}\left[{\text{H}}_{2}{{\text{O}}}_{2}\right]T\text{+1.09}\left[{\text{H}}_{2}{{\text{O}}}_{2}\right]t-\text{1.12 }Tt$$

The linear coefficient of temperature confirms the relevance of this factor, while interaction coefficients have a *p* value higher than 0.1, indicating the low significance in the model applied.

Similarly, total solids varied depending on the operating conditions applied. In some cases, TS did not appreciably change if compared to the untreated sludge; however, as a rule of thumb, under the most severe conditions applied, part of the organic matter was oxidized either towards volatile compounds or mineralized to carbon dioxide leading to a decrease in TS. Similar results were reported by Takashima and Tanaka (Takashima and Tanaka 2008) when applying different oxidative thermal treatments to an anaerobically digested sludge. TS was adjusted to the simplest significant model, the two-factor interaction leading to the expression (coded values):

3 $$\text{TS = 15.87}-\text{1.85}\left[{\text{H}}_{2}{{\text{O}}}_{2}\right]-\text{0.312 }T\text{+0.844 }t\text{ +3.28}\left[{\text{H}}_{2}{{\text{O}}}_{2}\right]T-\text{4.01}\left[{\text{H}}_{2}{{\text{O}}}_{2}\right]t-\text{2.77 }Tt$$

**Fig. 1:**
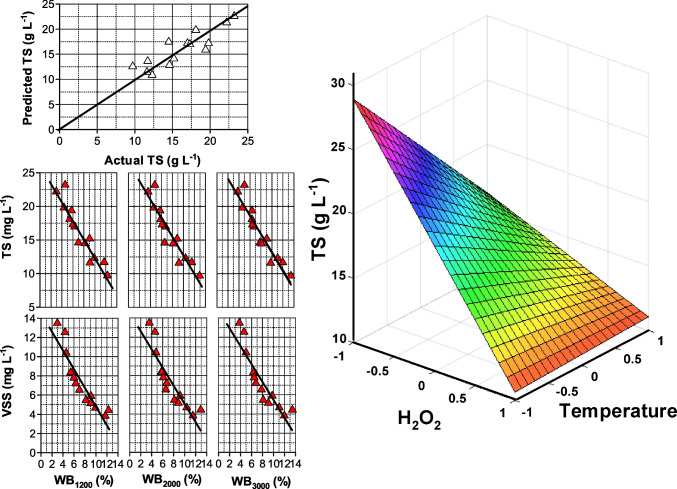
Thermal sewage sludge treatment in the presence of hydrogen peroxide. Modeling of total solid concentration by the two-factor interaction equation. **A** Comparison of calculated and experimental results. **B** Influence of hydrogen peroxide and temperature after 60 min of treatment. **C** Relationship between total solids (TS), water bound (WB), and volatile suspended solids (VSS)

In the above expression, it is seen that interaction factors are the most influencing coefficients while the coefficients corresponding to temperature and time present *p* values above 0.1, indicating that these two model terms are not significant. As suggested previously, the oxidation of sludge particles is favored when the most severe conditions are used. This is shown in Fig. 1A where predicted and calculated TS are displayed and the effect of temperature and hydrogen peroxide dose at the maximum time of 60 min is presented (Fig. 1B). When high H_2_O_2_ doses are used, temperature does not seem to significantly influence TS concentration; however, temperature is crucial if low amounts of the oxidant are applied. If H_2_O_2_ is sufficiently high, the concentration of radicals generated is warranted regardless of the temperature; in the case of low H_2_O_2_ load, temperature accelerates the decomposition of the peroxide to produce the required hydroxyl radicals. A linear relationship was found between this parameter (TS), water bound, and VSS (see Fig. 1C).

In all cases, partial oxidation of the sludge occurred (cell lysis) with the subsequent release of cell content and, as a consequence, increase in COD and TOC (Genç et al. 2002a). This is corroborated by the significant increase in dissolved COD and TOC before and after treatment. Hence, if comparing the organic content of s-EPS (no thermal extraction conducted) of raw and treated sludge, it can be observed that COD increased from roughly 150 mg L^−1^ to values in the range 3000–9000 depending on the operating conditions used while TOC varies from 70 to 90 mg L^−1^ to values in the interval 1000–3000 mg L^−1^. Similar results were reported by Genç and coworkers (Genç et al. 2002b) when treating a raw sludge by wet air oxidation in the presence and absence of catalyst or hydrogen peroxide. These authors reported an increase in TOC from 90 mg L^−1^ to values in the proximity of 2250 mg L^−1^. No significant differences were reported in runs completed at similar temperatures in the presence or absence of H_2_O_2_. Analogously, in this work, the most important parameter influencing the release of organic content was temperature. The higher the temperature, the higher the attack to sludge structure and COD, TOC, protein values (see Fig. S2 for trends).

Again, a two-interaction factor model was the optimum to check the relationship between variables and soluble COD with no thermal extraction. The expression in coded variables was

4 $$\mathrm s-\mathrm{COD}\;=\;6176.6\;-\;123.1\;\left[{\mathrm H}_2{\mathrm O}_2\right]\;+\;2091.4\;T\mathit\;+\;232.1\;t\mathit\;+\;806.8\;\left[{\mathrm H}_2{\mathrm O}_2\right]\;T\mathit\;\mathit-\mathit\;1268.2\;\left[{\mathrm H}_2{\mathrm O}_2\right]\;t\mathit\;-\;714.8\mathit\;Tt$$

**Fig. 2:**
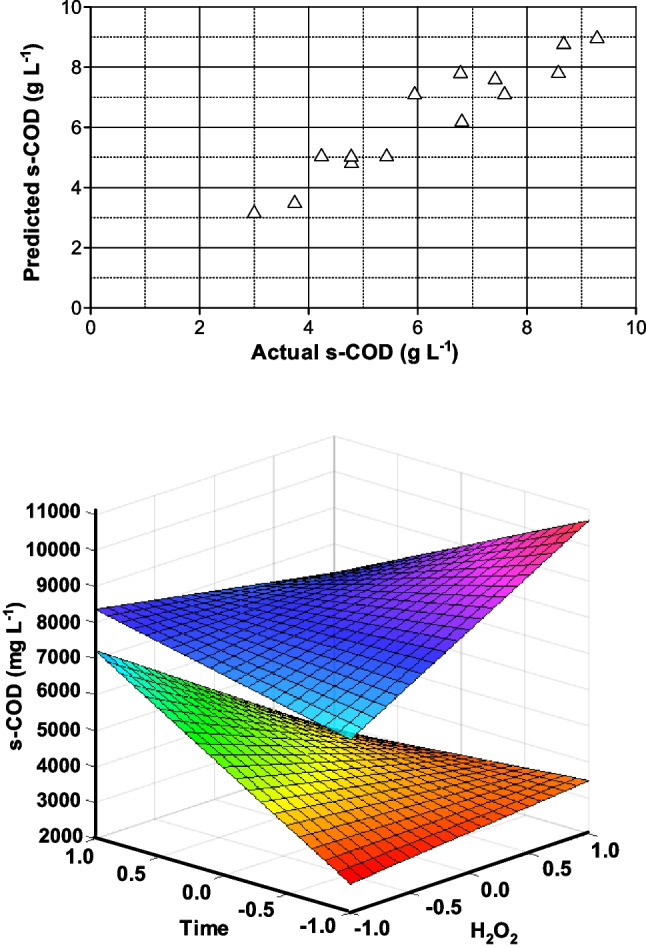
Thermal sewage sludge treatment in the presence of hydrogen peroxide. Modeling of soluble COD concentration by the two-factor interaction equation. Up: comparison of calculated and experimental results. Bottom: influence of hydrogen peroxide and time at 120 °C (bottom surface) and 180 °C (top surface)

The model *F* value is 11.86 implying that the model is significant (*R*^2^ = 0.9). There is only 0.13% that this *F* value could be attributed to noise. Coefficients shown in Eq. 4 reveal that temperature is the most influencing factor in COD together with the interaction between hydrogen peroxide dose and time. Figure 2 illustrates the model predictions and the influence of peroxide dose and time. The two surfaces shown in this figure correspond to the highest (top surface) and lowest (bottom surface) temperatures tested. As observed, when high temperatures are applied, hydrogen peroxide dose plays a more important role than time. It is assumed that H_2_O_2_ decomposition into radicals is relatively fast, and this is the reason why the reaction time does not significantly control the process. Contrarily, at low temperatures, the homolytic scission of the peroxide is the controlling stage and the system requires large times to be effective.

#### Dewaterability and filterability

A first approach to check any improvement in sludge filterability is the specific resistance to filtration or, alternatively, time to filter. Both parameters are intimately related and linearly correlated (*R*^2^ = 0.9). Results show different trends after treatment depending on the operating conditions applied. A simple linear model applied to SRF results reveals the influence of factors, temperature being the crucial parameter controlling the filterability of the treated sludge. Transformation of SRF to log_10_(SRF) was first applied to statistically analyze the results leading to the equation:

5 $${\text{log}}_{10}\text{(SRF) = 12.74}-\text{0.453}\left[{\text{H}}_{2}{{\text{O}}}_{2}\right]-\text{0.98 }T-\text{0.07 }t$$

**Fig. 3:**
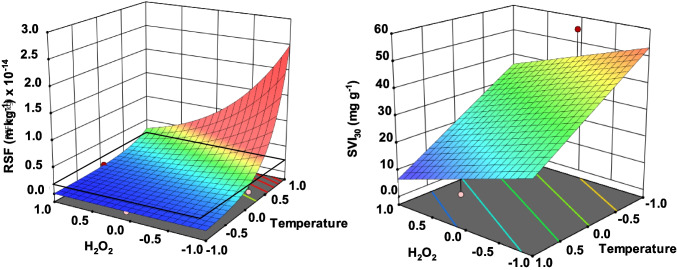
Thermal sewage sludge treatment in the presence of hydrogen peroxide. Linear modeling of soluble SRF (left) and SVI_30_ (right) as a function of temperature and hydrogen peroxide concentration after 60 min of treatment

Figure 3 (left) shows the effect of temperature which is more pronounced at low H_2_O_2_ loads. Similarly, the latter factor, i.e., hydrogen peroxide concentration, plays a more important role when temperature is low. Once more, radical generation rate seems to control the filterability of the treated sludge. At the sight of Fig. 3 (left) filterability of the sludge is not always guaranteed if compared to the value obtained for untreated raw sludge. Hence, an adequate combination of temperature and hydrogen peroxide is required to reduce SRF. Negative effects at low temperatures are also reported in other works associated to particle size and EPS structure modifications which can obstruct the filtration media. According to Bougrier and coworkers (Bougrier et al. 2008), there is a threshold temperature around 150 °C influencing sludge apparent viscosity, settleability, and dewaterability, likely related to protein solubilization and cell lysis. Similar results can be inferred from Fig. 3 where coded temperature 0 (= 150 °C) seems to be the frontier to improve SRF in comparison to untreated sludge. The presence of hydrogen peroxide seems to be essential. Liu et al. for instance (Liu et al. 2019) reported a negative effect in SRF of the thermal treatment in the range 20–170 °C. These authors claimed a decrease in the zeta potential associated to an increase in the ionization of proteins, humic substances, and polysaccharides. Consequently, repulsion between sludge particles occurs leading to poor dewaterability. The presence of hydrogen peroxide triggers the oxidation/destabilization of anionic substances avoiding the undesirable repulsion between particles. This was partially experienced by Liu and coworkers (Liu et al. 2022a) in the range of temperatures 135–170 °C.

Another important parameter in terms of sludge management is SVI_30_, which is linearly correlated to SRF. Once more, a simple linear expression in coded variables could model this parameter as a function of the three factors studied:

6 $${\text{SVI}}_{30}\text{ = 30.2}-\text{8.4}\left[{\text{H}}_{2}{{\text{O}}}_{2}\right]-\text{14.94 }T-\text{0.2 }t$$

Similar to SRF, temperature is the most influencing parameter; although in this case, logically, no negative results compared to raw sludge were obtained since the untreated sludge was collected from the recycling pipe, so this sludge was already settled. Time exerted a marginal effect in SVI_30_. Figure 3 right presents the influence of temperature and hydrogen peroxide load on this parameter. As the temperature approaches the lowest value (120 °C), SVI_30_ tends to the value obtained for the raw sludge (around 56 mL g^−1^). Different results were obtained by Genç and coworkers (Genç et al. 2002a) who found a 75% reduction in sludge volumetric index when applying a thermal treatment at 120 °C in the presence of roughly 0.035 M in H_2_O_2_.

A clear trend was observed between proteins, COD, and TOC of s-EPS and l-EPS and SVI_30_ (Fig. S3). The combination of temperature and radical oxidation leads to release of substances from the EPS structure (a part of cell lysis). Destruction of EPS structure triggers the improvement of sedimentation properties.

After completing the thermal sewage sludge treatment in the presence of hydrogen peroxide, the best conditions to obtain the precursors of adsorbents were selected. Hence, the main parameters considered were the final volumetric index and time to filter which are indicative of the easiness of sludge handling and water separation. Accordingly, operating conditions applied in run 5 (150 °C, 20 min reaction, and 0.03 M in H_2_O_2_) were chosen based on economic reasons if compared to other options (avoiding the highest temperature and considering the lowest reaction time tested). Treated sludge obtained under the aforementioned conditions was used to further carbonization and activation.

### Adsorbent synthesis and efficiency: preliminary experiments

As stated in the experimental section, several adsorbent preparation methods were compared; the main results of the obtained adsorbents are summarized in Table 2 and Table S1.

**Table 2:** Synthetized adsorbent characterization

Adsorbent	Carbonization *T* (°C)	Activation agent/*T* (°C)	Yield (%)	SA (m^2^ g^−1^)	APS (nm)	TPV (cm^3^ g^−1^)	% Carbon
DS	None	None	–	36	15.9	0.3	29.7 ± 0.2
S_400_	400	None	78	96	9.8	0.5	25.9 ± 0.5
S_700_	700	None	59	185	5.2	0.5	15.2 ± 0.3
S_KOH_	None	KOH/800	56	142	4.5	0.3	6.5 ± 0.1
S_NN_	None	NN/400	49	117	3.4	0.2	10.1 ± 1.0

*DS*:dehydrated sludge, *SA:* surface area, *APS:* averaged pore size, *TPV:* total pore volume

The synthesis of the adsorbents from dehydrated sludge led to a yield range in the interval 50–80% by weight of the initial raw material (dehydrated sludge). As a rule of thumb, the higher the carbonization temperature the lower the yield.

The XRF and elemental 

analysis (Table S1) revealed that the carbon content of the adsorbents is relatively low, varying between 6.5 and 25.9%, that is, equivalent to the loss of the raw carbonaceous material between roughly 12 and 80%. Loss of carbon is associated to any methodology implemented to produce adsorbents. Carbonaceous matter is destroyed in the creation of porosity, both by physical (Montes et al. 2022) and chemical activations (Kopyscinski et al. 2014). In any case, carbon destruction is not directly related to the porosity developed.

The inorganic content is formed mainly by Si, Fe, Al, Ca, P, Mg, and K, although other trace elements can be found such as S, Zn, Mn, Cu, Ti among others. These inorganics are present in the dewatering sludge, so they are not removed during adsorbent production treatments. According to XRF data, measured elements account for the 57% by weight of the DS sample. This percentage diminishes to approximately 50% when only thermal treatment (carbonization) is applied and 44% if chemical activation is completed.

**Fig. 4:**
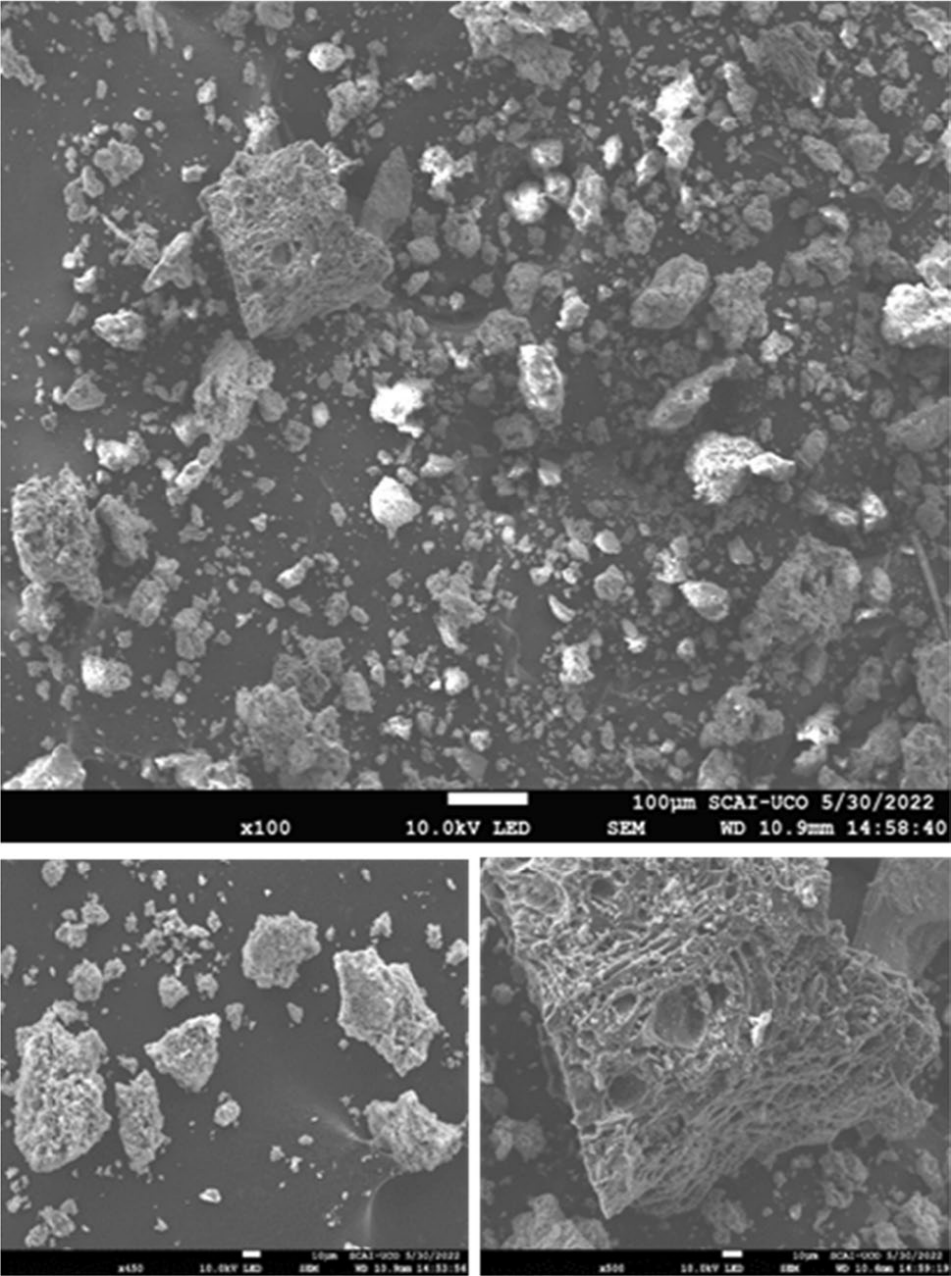
SEM image of the biosorbent S_NN_

**Fig. 5:**
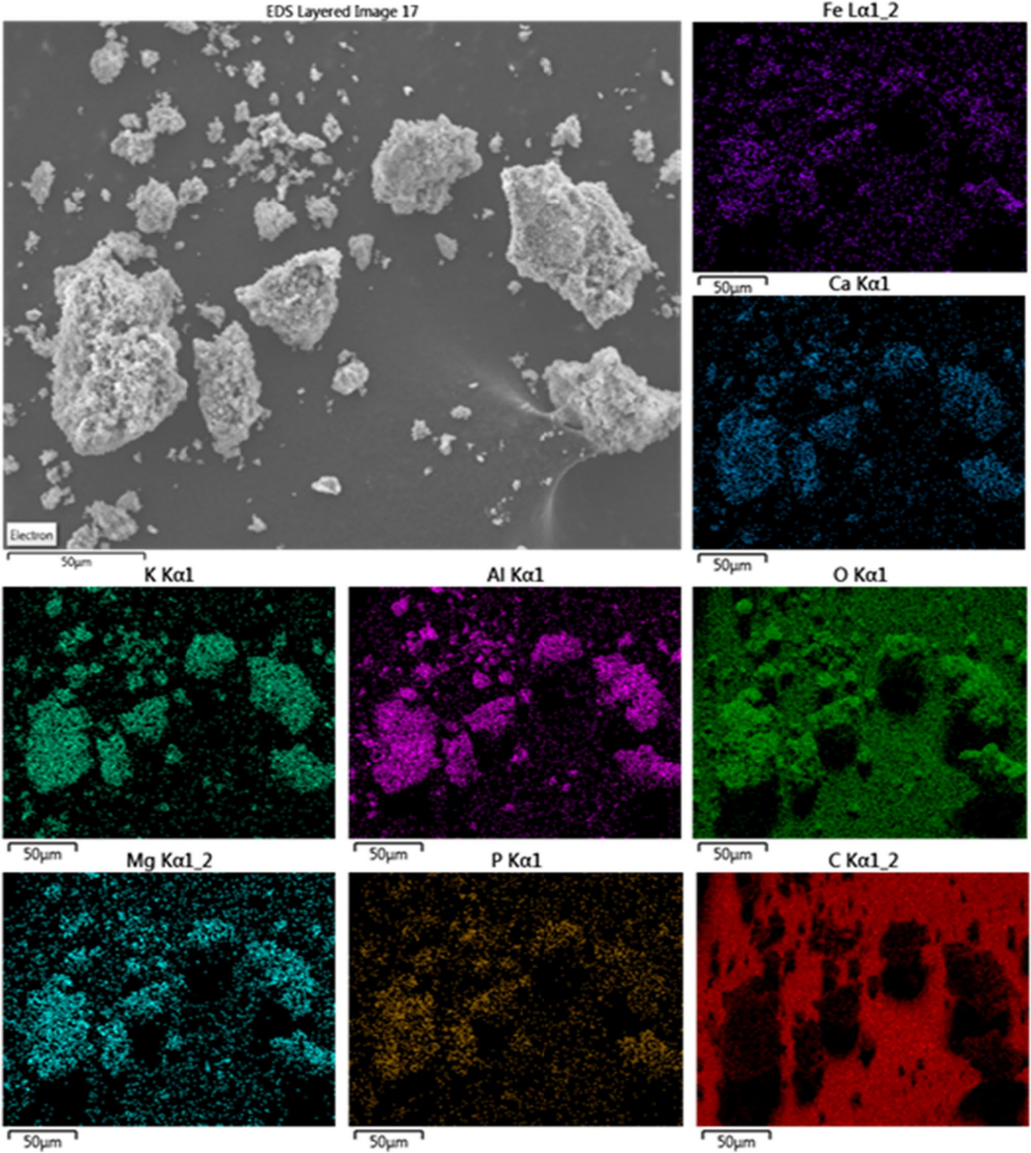
EDX image of the biosorbent S_NN_

SEM–EDX analysis reveals that the inorganic material forms particles between 2 and 200 µm (Fig. 4), separated from the carbonaceous fraction with a chemical composition quite homogeneous (Fig. 5).

It would be possible to remove a fraction of the inorganic content by acid washing. In the present study, this stage has not been considered for environmental sustainability reasons. Another option could be the implementation of an acid activation instead of the actual processes used. This could be a future research pathway. Water rinsing of adsorbents was carried out to check potential leaching of undesirable metals in solution. Leaching of inorganics was negligible from the third wash.

N_2_ adsorption experiments confirmed that all synthetized adsorbents led to type III isotherms. Nevertheless, the process carbonization-activation stages applied in this work did not result in the generation of adsorbents with large surface areas. The largest value was obtained after carbonization of DS at 700 °C, leading to a surface area of 180 m^2^ g^−1^, well below the common values reported for commercial activated carbons. The highest averaged pore size was obtained after carbonization at 400 °C with a value of 9.8 nm. Chemical surface was characterized by IR (not shown), resulting all of them in IR spectrum very similar, with bands centered at 996 cm^−1^ (Si–O-Si), 1635 cm^−1^ (amide band C = O vibration), 2920 and 2850 cm^−1^ (distinct aliphatic bands), and a broad band at 3400 cm^−1^ (OH) (Smidt and Parravicini 2009).

### Stability and adsorption capacity

In order to test the capacity of the adsorbents produced, stability and adsorption experiments were completed. Lixiviation runs revealed a significant amount of organic and inorganic matter leached after several consecutive water washing stages. A minimum of 10 washing steps were required to decrease the leaching of organic carbon under 2 mg L^−1^.

Lixiviation of either organic and inorganic species decreased as the synthesis temperature of carbonization/activation was increased. Inorganic leaching was especially significant when activation was made with KOH, suggesting the formation of carbonates under the operating conditions applied.

**Fig. 6:**
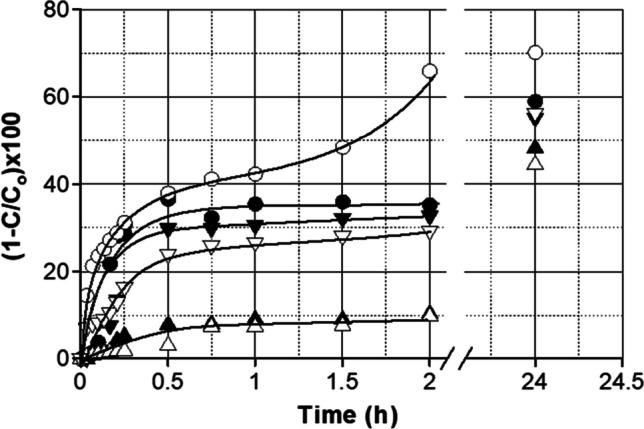
% Removal of 2 ppm (each) of ofloxacin (○●), phenol (△▲), and diuron (▽▼) on S_NN_ (1.0 g L^*−*1^ at 20 °C pH = 7). Open symbols = individual adsorption tests, solid symbols = mixture of the three compounds

Adsorption tests using phenol, ofloxacin, and diuron as model molecules are shown in Fig. 6. Some preliminary results completed with phenol demonstrated that S_NN_, with an adsorption capacity of approximately 6 mg of phenol per gram of adsorbent after 24 h, was the most effective bio-adsorbent in terms of parent compound removal. A first attempt to visualize the isotherm in this system was completed in equilibrium runs when the initial phenol concentration was varied in the range 2–25 ppm and 1 g L^−1^ of S_NN_. Several isotherm equations evaluated (Allahkarami et al. 2023, 2022; Javier Rivas et al. 2008) revealed that Langmuir type isotherm was the best fitting expression applied if the sum of the absolute error is considered (see Table 3).

**Table 3:** Isotherms relating adsorbed phenol in the biosorbent (*q*_*e*_ = mg phenol g^−1^ of biosorbent) and phenol equilibrium concentration in water (*C*_*e*_ = ppm of phenol)

Isotherm	Parameters				$\sum \left|{q}_{e}^{{\text{exp}}}-{q}_{e}^{\text{calc}}\right|$
Langmuir: ${q}_{e}=\frac{{q}_{m}{K}_{e}{C}_{e}}{1+{K}_{e}{C}_{e}}$	*q*_*m*_		*K*_*e*_		1.55
	6.6		2.26		
Temkin: ${q}_{e}=B{\text{ln}}({K}_{T}{C}_{e})$	*B*		*K*_*T*_		2.92
	1.04		59.4		
Freundlich: ${q}_{e}={K}_{F}{C}_{e}^{n}$	*K*_*F*_		*n*		3.97
	4.85		0.1		
Oswin: ${q}_{e}={K}_{\text{Osw}}{\left[\frac{{A}_{\text{Osw}}{C}_{e}}{1-{A}_{\text{Osw}}{C}_{e}}\right]}^{\lambda }$	*K*_Osw_	*A*_Osw_		*λ*	4.16
	7.46	0.013		0.1	
Caurie: ${q}_{e}={\text{exp}}({A}_{C}+{K}_{C}{C}_{e})$	*A*_*C*_		*K*_*C*_		6.35
	1.57		0.02		

Direct comparison with the performance of other adsorbents is normally risky since operating conditions and other adjacent circumstances (i.e., cost of the absorbent) should be considered. Hence, some authors (Allahkarami et al. 2023, 2022) report adsorption isotherms onto different activated carbons with phenol liquid equilibrium concentrations much higher than those used in this work; however, in the range of similar equilibrium concentrations, the capacity of sorbents seems to be comparable.

SNN was used in kinetic adsorption tests. Runs were carried out by using 2.0 ppm in each model contaminant (phenol, ofloxacin, and diuron). Adsorption experiments were conducted by using solutions of individual model compounds and the three compounds simultaneously.

Figure 6 reveals that the three substances are partially adsorbed from solution when they are treated individually (solid symbols). Phenol, due to its high solubility in water, was the most recalcitrant compound to be adsorbed with roughly 10% after 2 h. Contrarily, values in the proximity of 65 and 30% were experienced in the cases of ofloxacin and diuron, respectively. When the three substances were simultaneously adsorbed, observed trends were similar and the adsorption capacity remained almost unchanged after 24 h of treatment. The only difference observed was a reduction in the adsorption rate at the initial stages of the process.

At the sight of the results obtained from the performance of bio-adsorbents, the need of improving the synthesis process (both carbonization and activation) to generate solids with higher specific surface and enhanced adsorption capacity is recommended. The biosorbents obtained are not currently optimized concerning adsorption capacity. Therefore, a deeper analysis of the kinetics of the adsorption process is not pertinent at this point. A more thorough examination of the adsorption process is a future task, to be undertaken once the biosorbent capacity is optimized. In this subsequent work, kinetic equations and information on variable influence will be more meaningful.

## Conclusions

- Dehydration of activated sludge excess can be accomplished by thermal treatment in the presence of hydrogen peroxide.
- Hydrogen peroxide can decompose to generate hydroxyl radicals by homolytic scission. Radicals can trigger cell lysis changing the properties of the sludge.
- Temperature seems to be the crucial parameter to obtain acceptable results. Temperature has a double effect on radical generation. On the one hand, the kinetics of the H_2_O_2_ homolytic scission increases, and on the other hand, the inefficient recombination of radicals is also favored to produce oxygen and water.
- Optimization of temperature, hydrogen peroxide dose, and treatment time allowed to obtain a dehydrated solid that was converted to a bio-adsorbent by different carbonization-activation techniques.
- In preliminary experiments, it was shown that the biosorbent was capable of retaining/removing some model contaminants from water; however, a clear enhancement margin is required to make the whole process attractive from an environmental and economical point of view.
- Once the biosorbent synthesis process is optimized, a deeper study on the adsorption characteristics (capacity, kinetics, mechanism, etc.) is required.

### Supplementary Information

Below is the link to the electronic supplementary material.

Supplementary file1 (DOCX 177 KB)
