# Supplementary material for: Excess secondary sludge reuse by H2O2 thermal dehydration
Source: Environ Sci Pollut Res Int. 2024 Feb 28;31(15):23023–36. doi: 10.1007/s11356-024-32568-8 (PMC10997731; doi:10.1007/s11356-024-32568-8)
Supplement: Supplementary file 1 — Supplementary file1 (DOCX 177 KB) [file 11356_2024_32568_MOESM1_ESM.docx]

**Excess secondary sludge reuse: H_2_O_2_ treatment - thermal adsorbent synthesis**

Ana Udaondo^1,2^, Vicente Montes^*,2^, Olga Gimeno^1^, Francisco Javier Rivas^1^

^1^ Department of Chemical Engineering and Physical Chemistry, university institute of water, climate change and sustainability, University of Extremadura, Badajoz, Spain

^2^ Department of Organic and Inorganic Chemistry, university institute of water, climate change and sustainability, University of Extremadura, Badajoz, Spain

**SUPLEMENTARY INFORMATION**


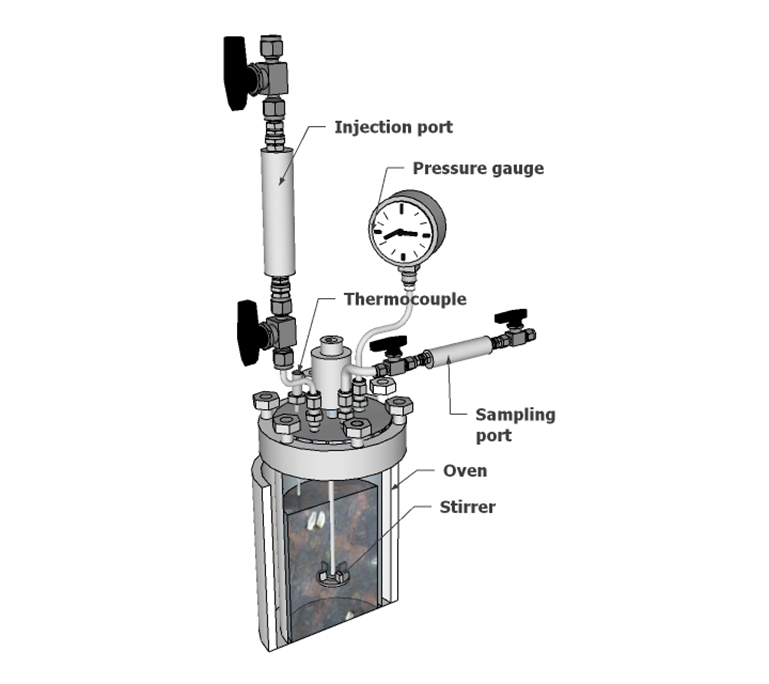


**Fig. S1.** Reactor used in the H_2_O_2_ treatment of secondary sludge.


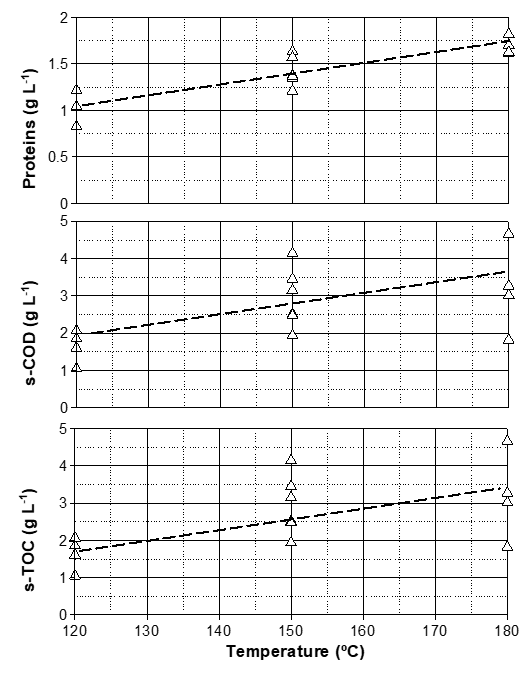


**Fig. S2.** Thermal sewage sludge treatment in the presence of hydrogen peroxide. Influence of temperature on the final concentration of up: Proteins. Middle: Soluble Chemical oxygen demand (s-COD). Bottom: Soluble Total Organic Carbon (s-TOC)


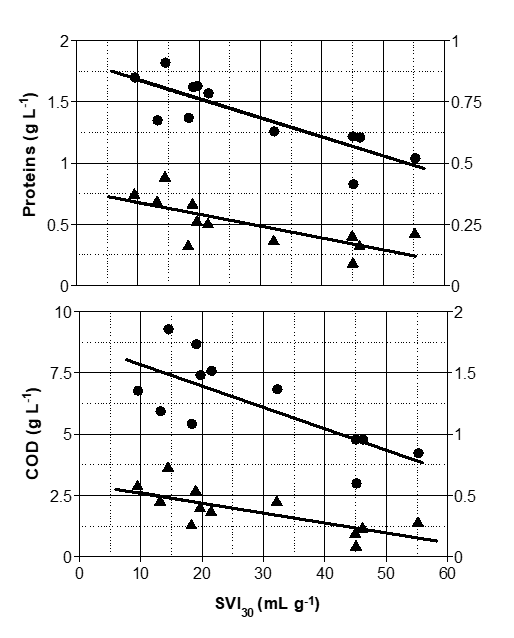


**Fig. S3.** Thermal sewage sludge treatment in the presence of hydrogen peroxide. Linear correlation between proteins and COD extracellular polymeric substances with sludge volumetric index after 30 min. ▲, s-EPS ●, l-EPS

**Table S1.** Elemental and XRF analysis pf synthetized adsorbents. Data in weight percentage

|  | **C** | **H** | **N** | **P** | **Fe** | **Al** | **Si** | **Ca** | **Mg** | **Ni** |
| --- | --- | --- | --- | --- | --- | --- | --- | --- | --- | --- |
| **DS** | 29.7 | 3.96 | 2.93 | 4.49 | 4.34 | 3.87 | 3.35 | 2.29 | 1.00 | - |
| **S_400_** | 25.9 | 2.03 | 2.11 | 4.65 | 4.24 | 4.17 | 3.90 | 2.41 | 1.23 | 0.02 |
| **S_700_** | 15.2 | 0.06 | 0.36 | 7.20 | 7.44 | 6.87 | 5.54 | 3.84 | 1.91 | - |
| **S_KOH_** | 6.5 | 1.96 | - | 1.15 | 8.44 | 6.62 | 8.30 | 5.04 | 2.46 | - |
| **S_NN_** | 10.1 | 0.18 | 1.19 | 6.36 | 7.27 | 5.79 | 5.87 | 3.46 | 1.51 | 0.02 |
|  | **K** | **S** | **Ti** | **Cl** | **Zn** | **Mn** | **Cu** | **Sr** | **Cr** |  |
| **DS** | 0.58 | 0.49 | 0.21 | 0.10 | 0.07 | 0.06 | 0.03 | 0.01 | - |  |
| **S_400_** | 0.64 | 0.18 | 0.19 | - | 0.06 | 0.06 | 0.02 | 0.004 | 0.02 |  |
| **S_700_** | 0.87 | 0.24 | 0.29 | - | 0.09 | 0.10 | 0.05 | 0.02 | 0.03 |  |
| **S_KOH_** | 2.27 | 0.34 | 0.39 | - | 0.08 | 0.11 | 0.09 | 0.02 | 0.04 |  |
| **S_NN_** | 1.06 | 0.10 | 0.31 | - | 0.13 | 0.07 | 0.05 | 0.02 | 0.02 |  |

DS=Dehydrated sludge
